# On the Influence of Noxious Effluvia on the Origin and Propagation of Epidemic Diseases

**Published:** 1853-04

**Authors:** 


### EPIDEMIOLOGICAL SOCIETY.

Mr. Grainger read a paper, of which the following is an abstract:

"*On the Influence of Noxious Effluvia on the Origin and Propagation of Epidemic Diseases.*—Although some diversity of opinion prevails among medical men in reference to epidemic disease, especially on the subject of contagion, all are agreed as to the noxious influence of overcrowding, defective ventilation, and other similar defects, prevalent in populous districts. It has occurred to me, that, without entering into the wide field connected with the nature and operation of noxious effluvia in general, it might not be altogether unprofitable if some elucidation of the facts which have fallen under my observation, both as regards the causation of, and exemption from, epidemic disease, were laid before the members of this Society. No one is more ready than myself to subscribe to the doctrine so well enunciated by my friend, Dr. Carpenter, in his valuable paper "On the Predisposing Causes of Epidemics," that it is not simply the collection and tabulation of facts, nor even mere empirical generalization, that will suffice; it is the principles and laws springing out of them which are demanded, if sanitary investigations are to be raised to the rank of a science. But fully recognising this as constituting the great aim and end of all these researches, and not forgetting the large amount of practical knowledge acquired of late years, it yet appears to me that there is abundant room and ample reason for elucidating evidence. Many points of prime importance to the public health as to matters of fact are still in much uncertainty. Doubts relating to agents assumed by sanitary inquirers to be deleterious still linger in the Profession, and by no means only among its least distinguished or influential members. The exact operation of animal effluvia, of a cesspool atmosphere, of excessive moisture,—conditions often combined in the miserable courts and alleys of our large towns,—is by no means fully ascertained. Extended inquiries of late years have abundantly proved that the same deleterious agents operate as predisposing causes in regard to the whole class of zymotic diseases; that what will develope the exciting cause of fever will also develope scarlatina, small-pox, diarrhœa, or cholera. So certain and notorious is this fact to those who practise among the poor, that before the outbreak of any epidemic, knowing where the predisposing causes are rife, they can foretell the precise localities where it will occur, nay, even name the alley or point to the exact house that will suffer. Such considerations have long induced me to conclude, that in regard to zymotic affections, the predisposing are infinitely more important than what are called the immediate or exciting causes. In regard to low fever, for example, it is certain that its efficient cause, the *materies morbi*, is never absent from London and other large towns; and yet it is rarely, many would say never, developed, unless there be superadded to it some predisposing cause. So true is this, that we not only daily see in the metropolis and elsewhere hundreds and thousands of persons living in the front streets exempt from typhoid fever, while the inhabitants of the wretched courts behind are scarcely ever free from it; but if by chance a given number of persons are planted in the very centre of an epidemic district, but freed from the predisposing causes of zymotic affections, they also, as a rule, will still escape."

"*On the Influence of Human Effluvia.*—According to my observation, the most injurious of all the causes operating on the diffusion of epidemic diseases, are the effluvia proceeding from the human body, particularly from the lungs and skin. The special deleterious agent consists of the effete, and, as it has been proved experimentally, highly putrescent organic matter, mingled with the expired air. That it is, when reintroduced into the living body, highly injurious, might be inferred from the very fact of the careful provision made by nature for its incessant elimination from the system. That it is small in amount is no objection to the intensity of its action; for, to the physiologist it is well known that a minute quantity of a powerful agent—the putrid matter introduced on the point of a needle in the inspection of a dead body, a single drop of concentrated prussic acid placed in the mouth of an animal, is sufficient to destroy life. It is in overcrowded bedrooms, in unventilated schools, workhouse dormitories, &c. that this effete matter taints the air, and, entering the blood, poisons the system. Although there is a great diminution in the amount of carbonic acid in the air evolved in the lungs, still the evil, quoad the development of fever, scarlatina, cholera, and so forth, depends on the organic, not the chemical products of respiration." The learned author referred to some experiments proving the truth of this assertion. He then continued: "It is, however, familiar to all practitioners, that human effluvia specially exhibit their poisonous influence when either multitudes of human beings are crowded together, or where a smaller number are placed in confined and unventilated sleeping-places. Many instances of the influence thus exerted on all kinds of epidemic disease, have come under my notice, but only a few illustrative examples can here be adduced. The following case illustrates the effect of overcrowding in respect to cholera. During the epidemic of 1849, the inmates of a reformatory establishment for young women suffered intensely from the pestilence, 40 out of a total of 96 being attacked, and 15, or rather more than 15 per cent., falling victims to the disease. Now, these poor sufferers were previously in perfect health; they were well fed, well clothed, and carefully attended; but the dormitories were low and much crowded; the windows, for the sake of seclusion, were partly closed up, thus greatly interfering with the ventilation. After a careful investigation, I could detect no other cause than this for the sudden outbreak occurring at a period when there was little cholera in the neighborhood. As regards the influence of overcrowding in the development of low fever, I may appeal to the experience of every medical practitioner whose duties call him much among the poor. It matters not whether we speak of the closely packed common lodging-house, of rows of houses built back to back, of the small, unventilated, and often single sleeping apartment of the mechanic, or of the ill-built cottages in rural districts, with their one bed-room, overhanging thatch and small lattice; wherever, either from the presence of numbers or the absence of ventilation, you have the fetid sickening air generated by human effluvia, there assuredly you will find fever. Although observed especially among the poor, fever, as it occurs in this country, is not especially dependent on poverty and destitution. Want may, indeed, aggravate the evil, and actual famine, as we unhappily saw a few years ago in Ireland, may give immense development to typhus; but that persons well fed, living in comfort, and strong in health, may suffer severely from low fever, is shown by a large experience. One of the best examples, perhaps, is furnished by the sailors belonging to the collier vessels frequenting the Thames. These men, as a body, are in the prime of life, robust and well fed; but as I found by examining many of these vessels, the place where they sleep—the forecastle—is excessively small and confined, and with this serious additional evil, that as the hatchway is usually flush with the deck, whenever there is much sea, it becomes necessary to close the hatchway, where the unfortunate sailors must be without any window, as if shut up in a close box. When, too, the vessels come to London, as only one man is required to keep watch at night, all the sailors are crowded at the same time into their closely-packed berths. Some years ago the attention of Mr. Busk, the distinguished surgeon of the Seaman's Hospital Ship, was attracted to the large number of typhus cases which were admitted. In 1841, they amounted to 147; in 1842, to 167. It appeared that of all the vessels in the Thames, the colliers furnished the most fever cases. In investigating this question I could detect no other cause than the polluted air these men must have breathed in the confined forecastle. That there is nothing connected with a sailor's mode of life to expose him to typhus, is proved by the experience of well-managed vessels, and, as one among the many proofs which might be adduced, I may mention that Mr. Clark, who has made ten voyages to India as surgeon in Messrs. Green's fine vessels, had never had a single case of typhus." The author, after referring to the very great improvement in the health of those of the working classes who inhabit the model lodging-houses erected in different parts of the town by the Society for the Improvement of the Dwellings of the Laboring Poor, and to the highly satisfactory working of the admirable

Act carried by the exertions of the Earl of Shaftesbury for controlling common lodging-houses, said that his own experience of the deplorable conditions of these abodes corroborated the statements of Capt. Hay; all tended to show that such pestilential places were the habitats of disease, and the cause of enormous expense to the rate-payers.—*London Med. Times and Gaz.*

